# Response to Letter to the Editor from de Zegher and Ibáñez: “Distinct Reproductive Phenotypes Segregate With Differences in Body Weight in Adolescent Polycystic Ovary Syndrome”

**DOI:** 10.1210/jendso/bvae076

**Published:** 2024-04-15

**Authors:** Laura C Torchen

**Affiliations:** Pediatric Endocrinology, Lurie Children's Hospital, Northwestern University, Chicago, IL 60611, USA

We would like to thank Drs. Ibanez and de Zegher for their comments on our recent manuscript published in *Journal of the Endocrine Society* [1, 2]. We agree that there is opportunity for such observations to translate to a more personalized approach to therapy in adolescents with polycystic ovary syndrome (PCOS). We would like to recognize the significant contributions Drs. Ibanez and de Zegher have made to the field of metabolic mechanisms and novel treatment approaches in adolescent PCOS.

We would caution readers against drawing conclusions regarding the prevalence of obesity in adolescents with PCOS based on the prevalence we report in our cohort. The CALICO database is not a population-based study but rather a retrospective database of clinical data from a number of academic medical centers across the United States. In fact, many of the patients included in CALICO received care in multidisciplinary PCOS clinics, which often have clinical support from dieticians and obesity medicine providers and specifically draw patients with obesity or other metabolic complications of PCOS. Therefore, we suspect the prevalence of obesity reported in our cohort significantly overestimates the population prevalence.

In terms of birthweight and its potential association with PCOS in our cohort, we had birthweight data available on 40 lean girls and 170 girls with obesity. There was a trend toward lower birth weight in the lean girls that did not reach statistical significance (lean girls 3.11 ± 0.57 vs obese girls 3.31 ± 0.62, kg, *P* = .07). Nine of the 40 lean girls had a birth weight of less than 2.8 kg, which is 10th percentile for a 40-week infant girl based on Centers for Disease Control and Prevention growth charts. Unfortunately, our database does not include gestational age, so we are unable to conclusively state the prevalence of small for gestational age status in this cohort.

In terms of treatment, we agree that therapies targeting the distinct mechanisms of PCOS would be the best approach, rather than a global recommendation for combination oral contraceptive pills as first-line therapy for all patients [3]. In fact, retrospective analysis of differential responses to a number of treatment approaches is a goal of our current grant funding the CALICO project (1R21HD109488). We hope preliminary data collected in this effort will allow for more prospective studies investigating individualized therapies based on distinct mechanisms, including ectopic fat and insulin resistance, which may result in the common PCOS end phenotype.

## Disclosures

The author has no disclosures to declare.
